# Guided Bone Regeneration Using a Novel Magnesium Membrane: A Literature Review and a Report of Two Cases in Humans

**DOI:** 10.3390/jfb14060307

**Published:** 2023-06-01

**Authors:** Marko Blašković, Ivana Butorac Prpić, Dorotea Blašković, Patrick Rider, Matej Tomas, Slavko Čandrlić, David Botond Hangyasi, Marija Čandrlić, Željka Perić Kačarević

**Affiliations:** 1 Department of Oral Surgery, Faculty of Dental Medicine Rijeka, University of Rijeka, Krešimirova 40/42, 51 000 Rijeka, Croatia; 2 Dental Clinic Blašković, Linićeva ulica 16, 51 000 Rijeka, Croatia; 3 Department of Dental Medicine, Faculty of Dental Medicine and Health Osijek, J.J. Strossmayer University of Osijek, Crkvena 21, 31 000 Osijek, Croatia; 4 Botiss Biomaterials GmbH, 15806 Zossen, Germany; 5 Department of Interdisciplinary Areas, Faculty of Dental Medicine and Health Osijek, J.J. Strossmayer University of Osijek, Crkvena 21, 31 000 Osijek, Croatia; 6 Department of Periodontology, Faculty of Dentistry, University of Szeged, Tisza Lajos krt. 64-66, H-6720 Szeged, Hungary; 7 Department of Anatomy, Histology, Embriology, Pathology Anatomy and Pathology Histology, Faculty of Dental Medicine and Health Osijek, J.J. Strossmayer University of Osijek, Crkvena 21, 31 000 Osijek, Croatia

**Keywords:** magnesium membrane, magnesium screws, biodegradable, dental implant, bone regeneration

## Abstract

Guided bone regeneration (GBR) is a common procedure used to rebuild dimensional changes in the alveolar ridge that occur after extraction. In GBR, membranes are used to separate the bone defect from the underlying soft tissue. To overcome the shortcomings of commonly used membranes in GBR, a new resorbable magnesium membrane has been developed. A literature search was performed via MEDLINE, Scopus, Web of Science and PubMed in February 2023 for research on magnesium barrier membranes. Of the 78 records reviewed, 16 studies met the inclusion criteria and were analyzed. In addition, this paper reports two cases where GBR was performed using a magnesium membrane and magnesium fixation system with immediate and delayed implant placement. No adverse reactions to the biomaterials were detected, and the membrane was completely resorbed after healing. The resorbable fixation screws used in both cases held the membranes in place during bone formation and were completely resorbed. Therefore, the pure magnesium membrane and magnesium fixation screws were found to be excellent biomaterials for GBR, which supports the findings of the literature review.

## 1. Introduction

Various pathological conditions can cause extensive changes in the hard and soft tissues of the oral cavity [1]. Additionally, the alveolar ridge undergoes large volume changes in horizontal and vertical dimensions after tooth extraction, which can complicate implant therapy and even, in some cases, make it impossible [2]. Insufficient volume for the alveolar ridge also affects the long-term outcome of implant therapy. Thus, successful long-term outcomes of implant therapy, including osseointegration, are achieved when implants are placed in the most biologically and prosthetically favorable position [1,3].

Guided bone regeneration (GBR) is the most well-documented surgical technique in the literature for horizontal and vertical alveolar bone regeneration [4]. Bone replacement in the vertical dimension is particularly challenging because there is no bone tissue at this level to help stabilize the graft material. In addition, it is also very challenging from a biological point of view, since the new bone and blood vessels must be created in a location that is distant from the existing bone tissue [5].

In GBR, various types of membranes are used as physical barriers [1,6]. Their main role is to seclude the bone defect from the rapidly migrating cells of connective and epithelial tissue. In this way, the much slower osteoprogenitor cells are allowed to fill the bone defect [7,8,9]. Membranes used in guided bone regeneration should have the following properties: biocompatibility, ability to integrate into the host tissue, easy clinical handling, volume stability, and appropriate mechanical and physical properties [10]. The simplest categorization of membranes used in GBR is into resorbable and non-resorbable membranes. The first generation of GBR membranes were non-resorbable and mostly made from polytetrafluoroethylene (PTFE) or expanded polytetrafluoroethylene (ePTFE). These membranes provide good biocompatibility and the ability to maintain space. However, as these membranes are non-resorbable, they must be removed in a second procedure after the bone regeneration treatment.

To avoid a secondary surgical procedure, a second generation of membranes were developed made of resorbable materials, which are now widely used for various clinical indications [6]. In addition, the use of resorbable membranes has been shown to be associated with a lower dehiscence incidence rate in comparison to non-resorbable membranes. [11]. Currently, collagen membranes are the most commonly used membrane type in bone and soft tissue regeneration. The collagen is mostly sourced from the dermis or pericardium of pigs or cattle. Their advantages are their resorption capacity and low immunogenicity and the possibility of incorporating drugs or additional biologically active components [12,13]. However, collagen membranes have an unpredictable resorption rate, which, in some cases, could negatively affect bone regeneration [6].

Magnesium metal has been investigated as an alternative resorbable material. Due to its many advantageous properties, such as mechanical strength [14], biocompatibility [15], degradability [16], and the fact that it is composed of trace elements already present within the human body [17], a new pure magnesium membrane and magnesium fixation screw have been developed. Previous publications have shown the biocompatibility and degradation of the magnesium fixation screws and pure magnesium membrane [18,19]. 

Recently, Elad et al. [20] published the results from using the magnesium membrane in a shield technique in a series of four cases. However, delayed implantation in humans after GBR with a magnesium membrane and magnesium fixation screw has not yet been reported. Therefore, the aim of this article was to summarize the current knowledge on magnesium membranes in dental regeneration procedures and to present the handling and regenerative potential of a novel magnesium membrane and fixation screws using two case reports of immediate and delayed implant placement.

## 2. Literature Search and Review of Current Knowledge on Magnesium Membranes

**Figure 1:**
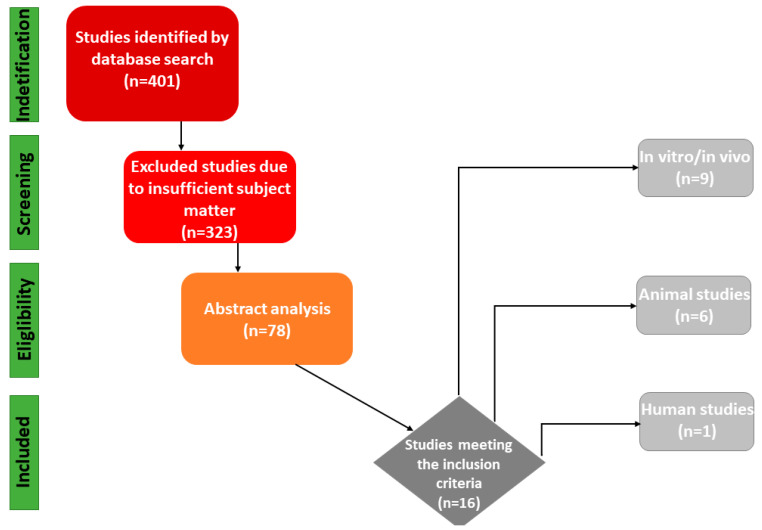
PRISMA flowchart of search results.

The literature search was completed in February 2023. The PubMed/MEDLINE, Scopus, and Web of Science databases were used for the literature search, with no restriction on the year of publication or type of publication. Thus, in vitro, in vivo, animal, and clinical studies; case reports or case series; and reviews on the topic of magnesium membranes in oral tissue regeneration were considered. The following keywords were used in combinations: “Guided Bone Regeneration”, “GBR”, “Tissue Regeneration”, and “Magnesium Membrane”. A total of 401 articles were reviewed, of which 323 were excluded for inappropriate subject matter. One investigator (I.B.P.) reviewed a total of 78 abstracts. Finally, a total of 16 reports met the criteria for the topic of interest (Figure 1). The selected papers were classified into the following categories: in vivo, in vitro, and animal studies (Table 1).

The studies reviewed showed heterogeneity in terms of the type of study, the type of animal models used, the follow-up period, the composition of the magnesium material, and the various outcome variables. Although a completely objective comparison of the studies was not possible, we would like to highlight some of the interesting and positive results in relation to magnesium.

The analysis of the mechanical properties of the magnesium membrane showed that the biomaterial is very stable and provides satisfactory shielding of the augmented area [19]. Numerous studies dealt with the resorption of magnesium membranes and the comparison with collagen membranes. A pure magnesium membrane was shown to degrade between 1 and 8 weeks after implantation [31]. Guo et al. [21] demonstrated that magnesium alloys WE43 and Mg3Gd corroded faster than Heal-All^®^. Modifying the surface of the magnesium mesh with Mg(OH)**_2_** resulted in delayed biodegradation. The study by Barbeck et al. also examined the effect of modifying the magnesium membrane that was treated with HF. The study showed higher cytocompatibility for the biomaterial with a HF coating and that the combination of magnesium and HF prevented the formation of gas cavities [28]. Several studies demonstrated the good biocompatibility of magnesium membranes [22,25,29]. Amberg et al. demonstrated the successful adhesion of human gingival fibroblasts to the membrane surface and showed how the migration rate changed as a function of Mg_2+_ and Ca_2+_ concentrations [23,24]. As mentioned earlier, Elad et al. reported the first use of the pure magnesium membrane in a completely new technique that was not previously possible with a resorbable membrane due to the lack of mechanical stability in the alternative options [20].

Overall, magnesium has been reported to provide the necessary characteristics for a barrier membrane. Since 2021, a pure magnesium membrane (NOVAMag^®^ membrane, botiss biomaterials GmbH, Zossen, Germany) has been available in Europe after receiving CE approval [31]. The magnesium membrane can be fixed with special fixation screws made of magnesium alloy (WZM211) with a MgF2 coating (NOVAMag^®^ fixation screw, botiss biomaterials GmbH, Zossen, Germany). In the next part of the paper, the application of the novel pure magnesium membrane and magnesium screws is demonstrated using two case reports with humans. 

## 3. Case Report One: GBR with Delayed Implant Placement

**Figure 2:**
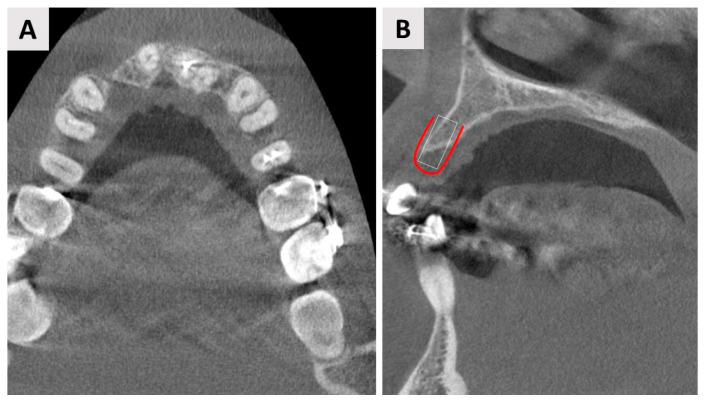
Axial (**A**) and coronal (**B**) sections from the CBCT before surgery showing significant bone loss in the horizontal and vertical dimensions. (**B**) The coronal section shows the planned direction for implantation (white rectangle) and the site designated for regeneration (red line).

After completion of orthodontic therapy, the patient was presented with the loss of tooth 12 (FDI notation system) and needed treatment to compensate for the functional and aesthetic absence of the tooth. Cone beam computed tomography (CBCT) analysis revealed bone deficiency in both the horizontal and vertical directions (Figure 2).

**Figure 3:**
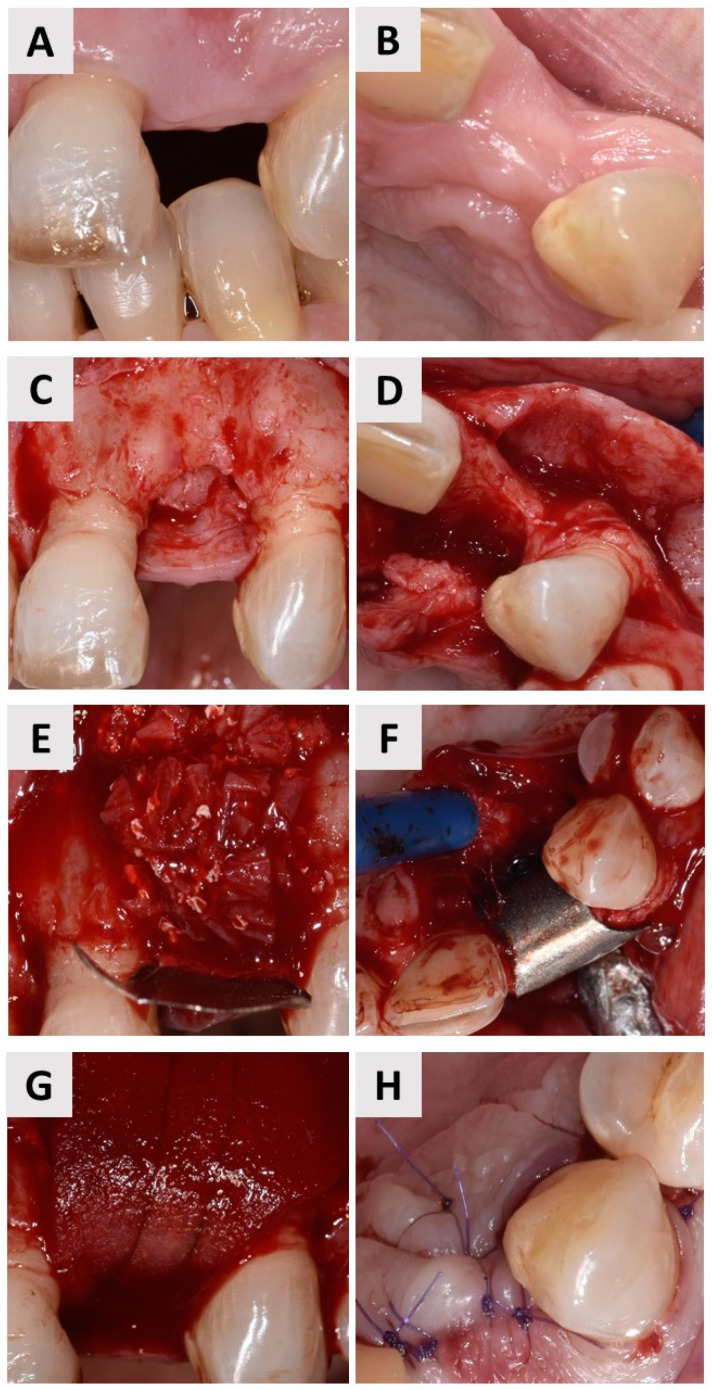
(**A**) Labial view of the edentulous space. (**B**) Proximal view of the edentulous space. (**C**) An intrasulcular incision was made on both adjacent teeth and a crestal incision was made at the augmentation site. Two releasing incisions were made in the labial mucosa to allow the complete mucoperiosteal flap to be elevated (labial view). (**D**) Elevated mucoperiosteal flap (occlusal view). (**E**) Application of the magnesium-based resorptive membrane, which was previously cut and shaped to the appropriate size using NOVAMag^®^ scissors (botiss biomaterials GmbH, Zossen, Germany). The membrane overlapped 3–4 mm from the defect margin. The defect was covered with a membrane and filled with a mixture of inorganic bovine bone and autologous bone. (**F**) A membrane was then carefully placed over the defect to secure the augmentation material with resorbable magnesium alloy screws. The positions of the drill holes on the membrane were marked with a NOVAMag^®^ sculptor. The screws were then inserted into the drilled holes. (**G**) The entire defect was covered with a xenogeneic collagen matrix (mucoderm^®^, botiss biomaterials GmbH, Zossen, Germany). (**H**) Primary wound closure was achieved with 6-0 sutures.

The patient was in good general condition and there was no contraindication for oral surgery. A prophylactic dose of oral antibiotics (Klavocin^®^ bid 875 mg + 125 mg, Pliva, Zagreb, Croatia) was administrated 1 h prior to surgery. Local anesthesia was applied (Ubistesin^®^ Forte 40 mg/mL + 0.01 mg/mL, 3M Deutschland GmbH, Seefeld, Germany), and the patient rinsed their mouth with 15 mL of a 0.2% chlorhexidine solution (Parodontax^®^ 0.2%, Brentford, London, UK) for 1 min. Deficits in the bone mass were clearly visible in the horizontal and vertical directions (Figure 3A,B). A complete mucoperiosteal flap was raised and the site intended for augmentation was exposed. Radiological findings of decreased bone volume were confirmed (Figure 3C,D). A bovine bone graft (cerabone^®^, botiss biomaterials GmbH, Zossen, Germany) and a small amount of locally harvested autogenous bone were mixed and used to augment the defect (Figure 3E). The completely novel magnesium membrane (NOVAMag^®^ membrane, botiss biomaterials GmbH, Zossen, Germany) was used to separate the defect site from the overlying soft tissue (Figure 3F). The edges of the membrane were shaped and flattened with the NOVAMag^®^ sculptor (botiss biomaterials GmbH, Zossen, Germany) to prevent perforation of the soft tissue. The membrane was secured to prevent slippage using resorbable magnesium screws (NOVAMag^®^ fixation screw, botiss biomaterials GmbH, Zossen, Germany) (Figure 3F). To achieve an optimal soft tissue profile, a soft tissue collagen graft (mucoderm^®^, botiss biomaterials GmbH, Zossen) was also placed over the membrane (Figure 3G). The entire defect was sutured in two layers using 6-0 sutures to achieve primary wound closure (Figure 3H). 

**Figure 4:**
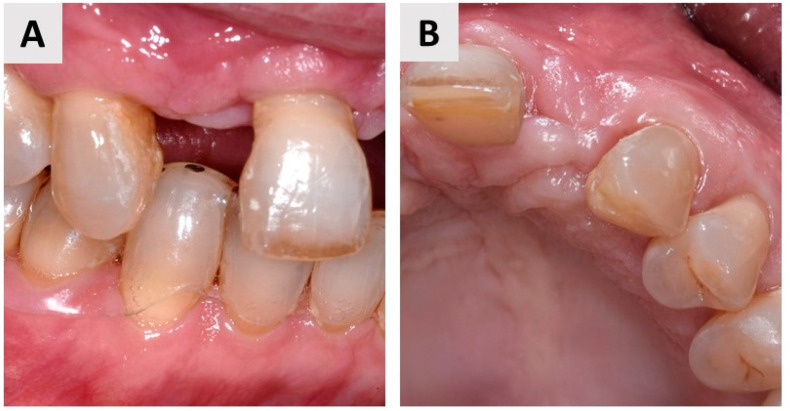
Labial (**A**) and occlusal (**B**) views of the regenerated site 3 months after augmentation.

The healing phase was uneventful, and the patient reported no side effects and no dehiscence of the biomaterial. The patient was followed up after 3 months, and the clinical outcome was satisfactory. Labial and oral mucosas were close together, and soft tissue could be seen to have regenerated (Figure 4A,B).

**Figure 5:**
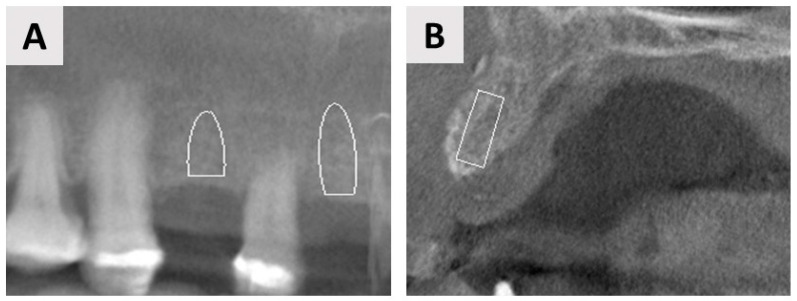
(**A**) A panoramic CBCT section shows bony healing in the vertical dimension. (**B**) Coronal CBCT section shows good bone regeneration in the vestibulo-oral dimension. White lines shows planned implantation site.

After a total of 5 months of healing, control CBCT was performed to evaluate the qualitative radiographic bone characteristics and to determine the type and size of the dental implant and the implant site in the regenerated area (Figure 5A,B).

## 4. Case Report Two: GBR with Immediate Implant Placement

**Figure 6:**
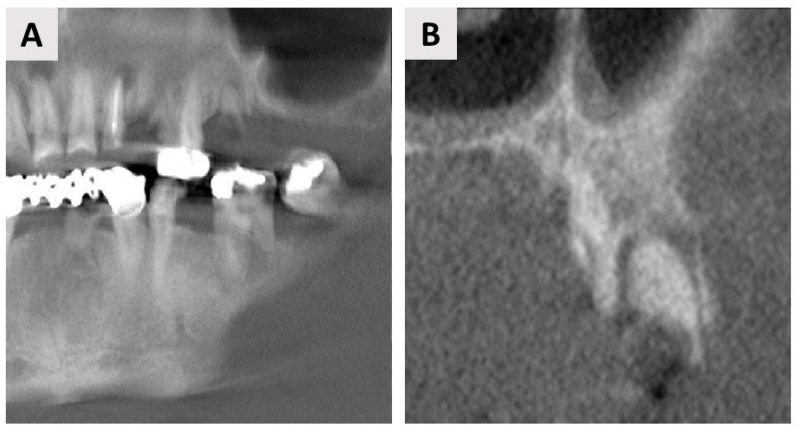
(**A**) A panoramic CBCT section shows the associated apical radiolucency of tooth 25 (FDI notation system). (**B**) The coronal CBCT section shows a closer look at tooth 25, which has a very thin buccal wall.

**Figure 7:**
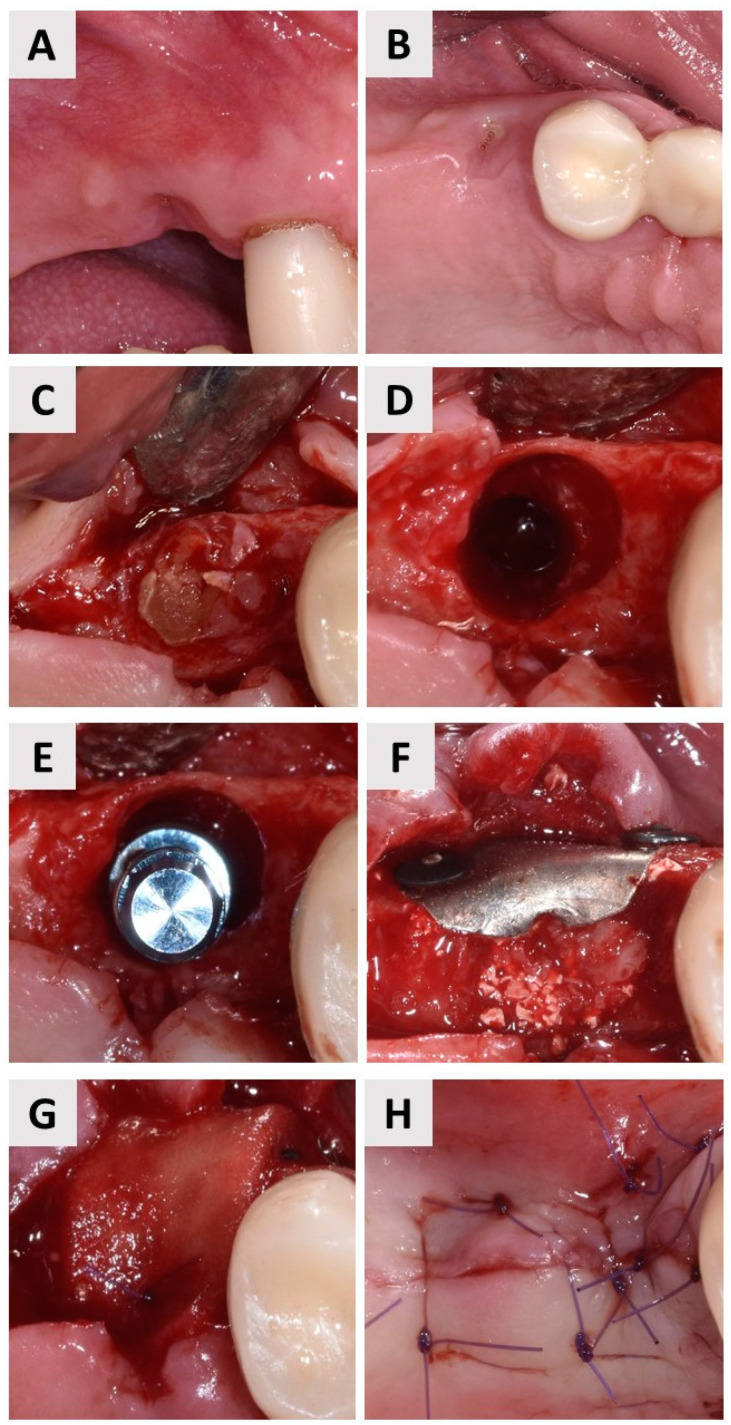
(**A**) Labial view of the residual root of the upper second premolar. (**B**) Occlusal view of the residual root of the upper second premolar. (**C**) Exposure of the residual root after flap elevation. (**D**) Preparation of the implant bed. (**E**) Insertion of the dental implant. (**F**) Magnesium membrane (NOVAMag^®^ membrane) was placed over the inorganic bovine bone and fixed with resorbable fixation screws (NOVAMag^®^ fixation screw, botiss biomaterials GmbH, Zossen, Germany). (**G**) Soft tissue graft (mucoderm^®^, botiss biomaterials GmbH, Zossen, Germany) for soft tissue augmentation, stabilized with sutures. (**H**) Primary wound closure was achieved using single 6-0 sutures.

The patient presented with a root remnant of tooth 25 (FDI notation system) (see Figure 6A,B and Figure 7A,B). CBCT confirmed a root remnant of the upper second premolar with a very thin buccal wall (Figure 6A,B). During treatment planning, it was decided to perform an immediate restoration with dental implantation and simultaneous bone augmentation according to the principles of the GBR technique. 

The preoperative protocol was the same as in the previously described case report, including antibiotic prophylaxis, administration of anesthesia, and mouth rinsing before the procedure.

An incision was made in the middle of the edentulous ridge and extended to the adjacent first premolar with an intrasuccular incision and a releasing flap. The mucoperiosteal flap was elevated to reveal the remaining root of the second maxillary premolar (Figure 7C). After atraumatic root extraction, the alveolus was curetted with a curette, and the remaining inflammatory tissue was removed. Following the manufacturer instructions, the implant site was prepared (Figure 7D) and the implant (Straumann BLT, Basel, Switzerland) was inserted (Figure 7E). A novel magnesium membrane (NOVAMag^®^ membrane, botiss biomaterials GmbH, Zossen, Germany) was placed on the buccal bone wall, and a layer of inorganic bovine bone (cerabone^®^, botiss biomaterials GmbH, Zossen, Germany) was placed between the membrane and the buccal wall (Figure 7F). Two magnesium screws (NOVAMag^®^ fixation screw, botiss biomaterials, Zossen, Germany) were used for membrane fixation (Figure 7F). A xenogeneic collagen matrix (mucoderm^®^, botiss biomaterials, Berlin, Germany) was cut, rehydrated in saline, and adapted to the augmented site Figure 7G). Finally, the edges of the mucoperiosteal flap were sutured in two layers to achieve primary wound closure (Figure 7H).

The patient was instructed about postoperative management. He was advised to use a 0.12% chlorhexidine mouth rinse after each meal, and a combination of amoxicillin and clavulanic acid (Klavocin 875 mg + 125 mg, Pliva, Zagreb, Croatia) was prescribed twice daily for 7 days to minimize the risk of infection. Three days after the operation, there was mild pain and slight swelling. Paracetamol was also prescribed for pain control. After two weeks, the sutures were removed. Clinically, healing was satisfactory, and no leakage of the biomaterial or exposure of the membrane was noted. The patient was referred for follow up and a CBCT scan in the next three months.

## 5. Discussion

Due to its excellent mechanical properties and biocompatibility, magnesium has been used for a long time for medical applications in fields such as cardiovascular surgery, musculoskeletal surgery, and general surgery [35]. To overcome the shortcomings of resorbable collagen membranes commonly used in GBR, a new resorbable magnesium membrane (NOVAMag^®^ membrane) has been developed. In this paper, we present a literature review and two case reports on the use of magnesium membrane in GBR. To the best of our knowledge, this is one of the first reports of GBR performed clinically using a completely resorbable magnesium membrane with immediate and delayed implant placement. 

Magnesium membranes provide improved properties, such as strength and dimensional stability, during wound healing without the need to reopen the surgical site for their removal [15]. The aforementioned mechanical properties allowed Elad et al. to develop a new technique, the magnesium membrane shield technique, which was described and applied to four of their clinical cases [20]. The handling of magnesium membrane is different from that of collagen. During implantation, the membrane becomes wet with saliva and blood in the moist environment of the oral cavity. This is a problem with collagen membranes as their mechanical properties are reduced and they are more prone to tearing during handling, whereas the magnesium membrane has high stability and low elasticity due to its metallic structure, and wetting does not affect handling [19,36,37]. Studies have shown that its tensile stress is much higher than that of collagen and polymeric membranes, which enables it to maintain its shape even after loading, such as after being covered by the soft tissue and during mastication [37,38,39]. Magnesium membranes have better potential to resist masticatory forces than collagen, even after 7 days under degrading conditions. Moreover, their ability to maintain shape during the critical healing period gives osteoprogenitor cells more time to proliferate at the site of the defect [19]. 

The biocompatibility, non-immunogenicity, and non-toxicity of the magnesium membrane has been demonstrated by a series of studies conducted by Rider et al. in accordance with internationally recognized standards for the evaluation of the biocompatibility of medical devices [19]. Magnesium metal and its alloys have excellent biocompatibility and are already used in established cardiovascular and orthopedic medical devices. When magnesium corrodes, magnesium ions are released as an oxidation product. Magnesium binds with water and forms a hydroxide layer that is susceptible to corrosion, especially in the presence of anions. The chloride ions present in the body fluids break down the hydroxide layer, allowing the corrosion process to continue [19].

Guo et al. came to an interesting conclusion when they compared the osteo-inductivity of Heal-All^®^ membrane and chitosan–magnesium (CS-Mg) membrane. Although no difference in the stimulation of bone formation was found between the two groups in vivo, in vitro tests showed that chitosan–magnesium (CS-Mg) membranes stimulated bone formation due to the magnesium component [25]. The good osteogenic effect of Mg^2+^ ions was also demonstrated by the research published by Wang et al. [33].

Steigmann proved that a membrane of pure magnesium met all the conditions for biocompatibility [29], and the good biocompatibility of magnesium alloys was demonstrated by Guo et al. in 2016 [21].

Dong et al. demonstrated that Mg^2+^ ions trigger uncontrolled generation of reactive oxygen species in bacteria, which increases oxidative stress within the bacteria and ultimately leads to bacterial damage. They also determined that magnesium derived from magnesium oxide nanoparticles promotes proliferation of osteoblasts [30].

The most important property of the new metal magnesium membrane is its resorption. Corrosion proceeds unevenly on the membrane surface, initially forming individual corrosion spots that, with time, spread over the entire membrane surface. Over a period of 8 weeks, most of the magnesium membrane corrodes, and during this time, bone formation occurs within the defect. Hydrogen gas also forms during this time, gently covering the soft tissue and serving as an additional barrier between the soft and hard tissues. In an animal study, Rider et al. described how the release of hydrogen gas decreases as corrosion of the pure magnesium membrane progresses, and gas formation ceases after 8 to 16 weeks [19]. In addition, magnesium salts are formed during membrane corrosion and retain the form of the metallic magnesium until they are replaced by newly formed bone [16,40,41,42].

Many studies have addressed the rate of degradation of magnesium-based membranes. To control the degradation rate, membrane surfaces have been modified with various coatings. For example, Shan et al. treated pure magnesium membranes with the coating MAO and demonstrated that MAO-treated pure Mg membranes performed better than uncoated pure Mg [32].

Byun et al. also modified the surface of a magnesium mesh by coating it with hydroxyapatite. This modification of the magnesium mesh gave good results in improving corrosion resistance. They also found that the coating HA mitigated the effects of magnesium mesh corrosion; namely, the formation of an alkaline environment and hydrogen gas [22].

In the study conducted by Barbeck et al. in which a magnesium mesh was treated with hydrofluoric acid, it was also found that the additional coating reduced the corrosion potential and the formation of gas cavities [28].

Wu et al. coated a magnesium mesh with a Ca- and P-containing layer, which slowed the decomposition of the magnesium mesh and improved bone regeneration [27]. Peng et al. also worked on a magnesium membrane coated with Ca-P and concluded that the coating not only reduced the rate of resorption but also the rate of hydrogen formation, thus improving bone regeneration [26].

With a Mg-Zn-Y-Nd alloy, Yan et al. reduced the degree of corrosion while achieving good biocompatibility and applicability [34].

In 2018 and 2019, Amberg et al. conducted studies on human gingival fibroblasts. They found that HGF migration depends on the concentration of Mg^2+^ and Ca^2+^, with the magnesium surface increasing the adhesion of HGFs and decreasing the rate of migration compared to resin and titanium surfaces [23,24].

In the cases reported in this article, we described the use of a magnesium fixation system (NOVAMag^®^ fixation screw, botiss biomaterials GmbH, Zossen, Germany). These were used to provide a completely resorbable system for each GBR procedure [18]. The fixation screws held the membrane in place and prevented micromovements that could negatively affect bone regeneration [43]. Undesirable micromovements can disrupt bone formation and promote the formation of fibrous tissue at the displacement site [44]. In order to reduce the resorption rate during the critical phase of wound healing, magnesium screws have a magnesium fluoride surface [18,45]. 

Alternative resorbable fixation systems, such as those based on polylactic acid (PLA) and polyglycolic acid (PGA), have been described as not fully biocompatible, mainly because of degradation products that may cause foreign body reactions or poor mechanical properties. In contrast, the degradation process of the magnesium alloy fixation system involves elements such as calcium, magnesium, phosphate, and fluoride, which are essential components of human tissue [46]. Therefore, the magnesium fixation system is fully biocompatible with the surrounding tissue.

The two cases reported represent one of the first reports on the application of a pure magnesium membrane and fixation screws in humans. The magnesium membrane and magnesium fixation screws proved to be easy to handle. In both cases, a collagen matrix (mucoderm^®^, botiss biomaterials GmbH, Zossen, Germany) was also applied to help improve soft tissue contours and achieve good aesthetic results. 

Overall, the cases supported the results of the literature search, as each case demonstrated satisfactory results in terms of clinical and radiological outcomes. Excellent mechanical properties and the ability to maintain mechanical stability during the healing phase are the main advantages of magnesium membrane. The membrane was gradually resorbed, and patients did not report side effects during healing. However, further studies with a larger number of patients are needed to fully demonstrate the regenerative potential of the pure magnesium membrane in humans.

## Figures and Tables

**Table 1:** Reports that met the criteria for the topic of interest.

Author	Title	Year	Study Type	Aim	Tested Biomaterial	Outcomes	Reference
Guo et al.	A preliminary study for novel use of two Mg alloys (WE43 and Mg3Gd)	2016	In vitro/in vivo	The aim of this research was to investigate two types of magnesium alloys (WE43 and Mg3Gd) compared with the Heal-All^®^ (Yantai Zhenghai Biotechnology Co., Shandong, China) membrane to determine whether the alloys can be used as biodegradable membranes	Magnesium alloy sheet (WE43 and Mg3Gd)	Degradation rate results for WE43 and Mg3Gd alloys showed no significant difference, but both Mg alloys corroded faster than Heal-All^®^ membrane. All three types of materials showed good biocompatibility	[21]
Byun et al.	The bioresorption and guided bone regeneration of absorbable hydroxyapatite-coated magnesium mesh	2017	In vivo (the rat calvarium)	The purpose of this study was to evaluate the absorption capacity of magnesium mesh coated with hydroxyapatite	Magnesium mesh coated with hydroxyapatite	A magnesium mesh coated with hydroxyapatite was shown to provide a reasonable process of bioresorption and bone reaction	[22]
Amberg et al.	Design of a migration assay for human gingival fibroblasts on biodegradable magnesium surfaces	2018	In vivo	The aim of this study was to investigate the migration behavior of human gingival fibroblasts on the surface of magnesium	The pure magnesium membrane	The results of this study showed that human gingiva fibroblasts adhered to and formed confluent layers on a precorroded magnesium membrane surface; however, the cells migrated more slowly over the surface compared to plastic and titanium	[23]
Amberg et al.	Effect of physical cues of altered extract media from biodegradable magnesium implants on human gingival fibroblasts	2019	In vivo	The aim of the study was to investigate the effects of minerals such as Mg^2+^, Ca^2+^, H_2_ and increased osmolality, as well as the effects of magnesium extracts, on human gingival fibroblasts (HGFs) in terms of their migration, proliferation, and viability	Pure magnesium membrane (NovaMag^®^ membrane, botiss biomaterials, Zossen, Germany, Mg purity: 99.95%)	The migration rate of HGFs tends to slow down when the ratio of Mg^2+^ and Ca^2+^ changes because the concentration of Mg^2+^ increases and the concentration of Ca^2+^ decreases near the corroding magnesium implant	[24]
Guo et al.	Biocompatibility and osteogenic activity of guided bone regeneration membrane based on chitosan-coated magnesium alloy	2019	In vitro and in vivo (rabbit calvaria)	The objective was to evaluate the performance of chitosan–magnesium membrane prepared with a specific protocol for the needs of the study and to compare the results with the commercially available membrane Heal-All^®^ (Yantai Zhenghai Biotechnology Co., Shandong, China)	Composite chitosan–magnesium (CS-Mg) membrane fabricated by dip-coating Mg alloy into chitosan solution	In vitro: CS-Mg had a suitable degradation rate and similar cell adhesion and cytocompatibility as commercially available membrane In vivo: new bone formation was good in both groups compared to the blank control. There were no significant differences between the CS-Mg and Heal-All^®^ groups (*p* > 0.1)	[25]
Peng et al.	Mg-based absorbable membrane for guided bone regeneration (GBR): A pilot study	2019	In vitro/in vivo	The aim of this study is to investigate microstructural characteristics and perform electrochemical testing, immersion testing, fluorescent labeling analysis, and histopathological evaluation of magnesium membrane coated with calcium phosphate	Calcium phosphate-coated Mg membrane	The Ca–P coating increased the corrosion resistance of the Mg membrane in vitro and in vivo and achieved better results than pure Ti membranes in terms of membrane duration	[26]
Wu et al.	Surface modification of pure magnesium mesh for guided bone regeneration: In vivo evaluation of rat calvarial defect	2019	In vivo (rat calvaria defect)	The aim of this research was the surface modification of pure magnesium mesh using plasma electrolytic oxidation and hydrothermal treatment	Magnesium mesh with a protective layer that mainly consisted of Mg (OH)_2_ with amorphous calcium phosphate	Biodegradation of the magnesium mesh was found to be significantly retarded, and surface modification of Mg could also improve volume and bone density of the calvarial defect compared to that of pure Mg mesh	[27]
Barbeck et al.	Degradation, bone regeneration and tissue response of an innovative volume stable magnesium-supported GBR/GTR barrier membrane	2020	In vitro/in vivo	The aim of this research was to investigate a new bioresorbable hydrofluoric acid (HF)-treated magnesium (Mg) mesh in native collagen membrane for stable-volume situations	Hydrofluoric acid (HF)-treated magnesium (Mg) mesh	In vitro: Mg treated with HF showed higher cytocompatibility. Histopathologically, HF-Mg prevented gas voids while untreated Mg showed partially significantly more gas voids and fibrotic tissue reaction. In vivo: bone regeneration was not significantly different between all groups	[28]
Steigmann et al.	Biocompatibility and immune response of a newly developed volume-stable magnesium-based barrier membrane in combination with a PVD coating for guided bone regeneration (GBR)	2020	In vitro and in vivo (the subscapular region of BALB/c mice)	The aim of the study was to analyze a new approach based on ion implantation (II) with a PVD coating for passivation of newly developed Mg membranes for GBR/GTR procedures	Mg membranes were passivated by ion implantation in an argon atmosphere followed by PVD treatment using a specially designed coating system	In vitro: untreated and PVD-coated membranes were not cytocompatible as static conditions could not be used for magnesium in vitro tests. Both types of membranes showed good biocompatibility in in vivo studies	[29]
Rider et al.	Biodegradable magnesium barrier membrane used for guided bone regeneration in dental surgery	2021	In vitro and in vivo (Yucatan minipigs)	The aim of this study was to analyze chemical and mechanical properties, in vitro corrosion, and in vivo corrosion in Yucatan minipigs	NOVAMag^®^ membrane (botiss biomaterials, Zossen, Germany)	The magnesium membrane exhibited mechanical stability that allowed satisfactory shielding of the augmentation site. The magnesium membrane was completely resorbed, and bone healing was completed before current standards for treating patients with a second surgical procedure	[19]
Dong et al.	Antimicrobial and pro-osteogenic coaxially electrospun magnesium oxide nanoparticles-polycaprolactone/parathyroid hormone-polycaprolactone composite barrier membrane for guided bone regeneration	2022	In vitro/in vivo	The goal was to produce an antibacterial and pro-osteogenic coaxial electrospun membrane for guided bone regeneration (GBR) from nanofibers to meet the complicated phasic requirements of the GBR process	GBR membrane of coaxially electrospun nanofibers with parathyroid hormone (PTH) encapsulation in the central layer and magnesium oxide nanoparticles (MgONP) in the shell layer (MgONP-PCL/PTH-PCL)	MgONP-PCL/PTH-PCL showed remarkable antibacterial potential through the release of MgONPs. It was also found that the incorporation of MgONP significantly prolonged the release of PTH. High-dose PTH promotes membrane pro-osteogenicity to improve the efficiency of bone regeneration in the presence of MgONP	[30]
Rider et al.	Analysis of a pure magnesium membrane degradation process and its functionality when used in a guided bone regeneration model in beagle dogs	2022	In vivo (beagle dogs)	The aim was to evaluate the degradation process and the potential for tissue regeneration of a pure magnesium membrane and to compare it with the commonly used collagen membrane	NOVAMag^®^ membrane (botiss biomaterials, Zossen, Germany)	The greatest rate of magnesium membrane degradation was seen between 1 and 8 weeks after implantation and continued until week 16. New bone formation was similar in both groups, suggesting that magnesium membrane may be an alternative to collagen membrane	[31]
Shan et al.	Degradable pure magnesium used as a barrier film for oral bone regeneration	2022	In vitro and in vivo (cranial parietal bone of experimental rabbits)	The aim was to conduct electrochemical tests, immersion tests, and in vivo tests to investigate the potential of the magnesium membrane as a barrier membrane	Pure Mg membrane surface treated with micro-arc oxidation (MAO)	In vitro: experimental results showed that the corrosion resistance of MAO-treated pure Mg membrane was better than that of uncoated pure Mg, and cell experiments showed no cytotoxicity. In vivo: MAO-Mg membrane showed better biological activity than pure Ti membrane in the early stage of implantation and good bone regeneration ability	[32]
Wang et al.	Photocrosslinkable Col/PCL/Mg composite membrane providing spatiotemporal maintenance and positive osteogenetic effects during guided bone regeneration	2022	In vitro/in vivo	The aim was to design a photocrosslinkable collagen/polycaprolactone methacryloyl/magnesium (Col/PCLMA/Mg) composite membrane that provides a spatiotemporal support effect after photocrosslinking	A photocrosslinkable collagen/polycaprolactone methacryloyl/magnesium (Col/PCLMA/Mg) composite membrane	Col/PCL and Col/PCL/Mg membranes showed a much higher elastic modulus and lower swelling rate than Col membranes, and there were no differences in cell biocompatibility between groups. Col/PCL and Col/PCL/Mg membranes had lower degradation rates than Col membranes. Col/PCL/Mg groups showed improved osteogenic ability compared to Col groups	[33]
Yan et al.	Feasibility and efficacy of a degradable magnesium-alloy GBR membrane for bone augmentation in a distal bone-defect model in beagle dogs	2022	In vivo (beagle dogs)	The aim of the study was to investigate the effectiveness and feasibility of guided bone regeneration using a degradable magnesium alloy for the healing of bone defects after tooth extraction	Degradable Mg alloy regeneration membrane (MAR-Gide (MG))	Mg alloy membrane regeneration did not increase the prevalence of infection, dehiscence, or subcutaneous emphysema compared to those who used Bio-Gide. It also showed good biocompatibility and clinical applicability	[34]
Elad et al.	Application of biodegradable magnesium membrane shield technique for immediate dentoalveolar bone regeneration	2023	Clinical case series (humans)	The aim was to demonstrate the first clinical usage of a magnesium metal membrane in a shield technique	NOVAMag^®^ membrane (botiss biomaterials, Zossen, Germany)	In all presented clinical cases, there was good regeneration of bone tissue and healing of soft tissue	[20]

## Data Availability

The data presented in this article are available on request from the corresponding author.
